# Representation of Glossy Material Surface in Ventral Superior Temporal Sulcal Area of Common Marmosets

**DOI:** 10.3389/fncir.2017.00017

**Published:** 2017-03-17

**Authors:** Naohisa Miyakawa, Taku Banno, Hiroshi Abe, Toshiki Tani, Wataru Suzuki, Noritaka Ichinohe

**Affiliations:** ^1^Department of Ultrastructural Research, National Institute of Neuroscience, National Center of Neurology and PsychiatryKodaira, Japan; ^2^Ichinohe Neural System Group, Laboratory for Molecular Analysis of Higher Brain Function, RIKEN Brain Science InstituteWako, Japan

**Keywords:** vision, material, visual category, primate, monkey, superior temporal sulcus, multi-electrode array

## Abstract

The common marmoset (*Callithrix jacchus*) is one of the smallest species of primates, with high visual recognition abilities that allow them to judge the identity and quality of food and objects in their environment. To address the cortical processing of visual information related to material surface features in marmosets, we presented a set of stimuli that have identical three-dimensional shapes (bone, torus or amorphous) but different material appearances (ceramic, glass, fur, leather, metal, stone, wood, or matte) to anesthetized marmoset, and recorded multiunit activities from an area ventral to the superior temporal sulcus (STS) using multi-shanked, and depth resolved multi-electrode array. Out of 143 visually responsive multiunits recorded from four animals, 29% had significant main effect only of the material, 3% only of the shape and 43% of both the material and the shape. Furthermore, we found neuronal cluster(s), in which most cells: (1) showed a significant main effect in material appearance; (2) the best stimulus was a glossy material (glass or metal); and (3) had reduced response to the pixel-shuffled version of the glossy material images. The location of the gloss-selective area was in agreement with previous macaque studies, showing activation in the ventral bank of STS. Our results suggest that perception of gloss is an important ability preserved across wide range of primate species.

## Introduction

Knowing what material an object is made from is critically important in judging the quality and purpose it may serve, or in identifying semantic category the object belongs to. Surface information, such as reflectance and texture pattern can often provide cues for fast and effortless perception of the material identity and quality (Adelson, 2001). Recently, functional imaging studies in human (Cant and Goodale, 2007; Hiramatsu et al., 2011) and non-human primates (Goda et al., 2014) have identified that visual information related to the material of the object is processed in the ventral visual stream, changing from image-based to perception-related information as it is carried rostrally along the temporal lobe (Hiramatsu et al., 2011). Electrophysiological research in macaque monkeys has investigated the detailed property of neurons selective to surface texture in V4 (Okazawa et al., 2015) and surface glossiness in superior temporal sulcus (STS; Nishio et al., 2012, 2014). However, precise cortical mapping for these surface properties has been difficult partially due to the intrasulcal localization of such neurons.

The common marmoset (*Callithrix jacchus*), a New World monkey, is one of the smallest primate species. An important advantage of the marmoset is its lissencephalic (smooth) brain structure, making it an ideal target for precise cortical mapping (Suzuki et al., 2015b) and *in vivo* study of cortical connections (Ichinohe et al., 2012; Suzuki et al., 2015a). It has become a popular non-human primate model of the visual system (Solomon and Rosa, 2014; Mitchell and Leopold, 2015) due to their high visual and cognitive abilities, demonstrated by its performance on visual discrimination (Roberts et al., 1990), tool use (Yamazaki et al., 2011), visual fixation in head-restrained condition (Mitchell et al., 2014; Mitchell and Leopold, 2015) and evaluation of social interaction between others (Kawai et al., 2014). Progress in the past couple of years has enabled functional imaging and ECoG (Hung et al., 2015a,b), and chronic electrophysiology in the STS and ventrolateral prefrontal cortex awake conditions (Suzuki et al., 2015a).

As marmosets are known to rely mainly on their vision when foraging for food, they are likely to possess a highly developed visual material perception system utilized for judging the identity and quality of the targeted food from its surface appearance (Perini et al., 2009).

In this study, we investigated whether there are neurons selective to the type of material in the marmoset ventral STS region, and whether there are functional structures for neurons selective to material representation. We recorded from up to 128 sites in high density using multi-contact silicone electrodes while presenting visual stimuli that vary in three different shapes and resembles surface property of eight different material or appearance types. We also presented the animal with a stimulus set that resembles the original set, but with each pixel shuffled. Neuronal activities were defined as material-selective when they were sensitive to the type of material (two-way analysis of variance (ANOVA) for material appearance and shape), and had a preference for the original object images over the shuffled images. We found that in a ventral part of the fundus of the superior temporal area (FSTv), there is a cluster of neurons that have selectivity to the glossy image categories, such as glass and metal.

## Materials and Methods

Experiments were performed using four adult common marmosets (weighing 300–450 g). This study was approved by the Experimental Animal Committee of the National Center of Neurology and Psychiatry. The animals were treated in accordance with the “Guiding Principles for the Care and Use of Animals in the Field of Physiological Science” of the Japanese Physiological Society.

### General Surgical Procedures

We followed Bourne and Rosa’s general guideline for the marmoset experiments (Bourne and Rosa, 2003). Anesthesia was induced with an intramuscular injection of ketamine hydrochloride (Ketalar, 25 mg/kg) following an intramuscular injection of atropine sulfate (0.15 μg/kg) for saliva suppression. Animals were artificially ventilated with a mixture of nitrous oxide (70%), oxygen (30%), and maintained in anesthesia with isoflurane (1%–2%) during the surgery. Electrocardiograms, expired CO_2_ and rectal temperature were monitored continuously throughout the experiment. The animals were placed in a stereotactic apparatus and a head holder was fixed onto the skull with anchor screws and dental resin. Animals were removed from the stereotactic frame and fixed by the head holder for a further surgery. A recording chamber was fixed with dental resin after detaching and lowering the temporal muscle. Craniotomy and duratomy inside the chamber exposed the temporal cortex around the STS.

### Electrophysiological Recordings

For electrophysiological recordings, anesthesia was switched from isoflurane to intravenous infusion of remifentanil (Ultiva, 0.1 μg/kg/min) and rocuronium bromide (Eslax, 13 μg/kg/min). Pupil was fully dilated with topical tropicamide (0.5%) and phenylephrine hydrochloride (0.5%) before the recordings. Electrodes were inserted in reference to two sulcal landmarks: the STS and the lateral sulcus. A micromanipulator lowered a 32 or 128 channel multicontact linear-array electrode (NeuroNexus, Ann Arbor, MI, USA), which contained 4 or 16 shanks with 400 μm horizontal spacing. A penetration took 15–20 min, and a resting period after the penetration took 30–60 min. Electrode shanks were 5 mm in length and each shank had eight electrode contacts (impedance, ~1 MΩ at 1 kHz) covering 1400 μm from the tip with 200 μm vertical spacing. We inserted the electrode perpendicular to the cortical surface ventral to the posterior end of STS. Neural signal was fed into a TDT signal processing system (RZ2, Tucker-Davis Technologies, Alachua, FL, USA), band-pass filtered between 300 Hz and 5 kHz, and stored at 24 kHz resolution together with the digital time stamps of the visual stimulus presentation (onset and offset). Time points at which the waveform fell below −3.5× the standard deviation (SD) of the signal were stored as multiunit time stamps. After the electrophysiological recordings, subgroup of shanks in the 4-shank (32-channel) array, or the 16-shank (128-channel) array were marked with microneedles smeared with 1,1′-dioctadecyl-3,3,3′,3′-tetramethylindocarbocyanine perchlorate (DiI; D282, Thermo Fisher Scientific, Waltham, MA, USA).

### Histology and Area Demarcation

Animal 1 was sacrificed immediately after the recordings. Animals 2–4 were recovered and used in other experiments before scarification. To sacrifice, the animals were overdosed with sodium pentobarbital (Somnopentyl, 75 mg/kg i.p.) After tail-pinching to confirm that animals are under deep anesthesia, they were perfused intracardially with 0.1 M PBS (pH 7.4) and 4% paraformaldehyde (PFA) sequentially. Brain block was removed from the animal and put under post fixation in 4% PFA overnight. PFA was replaced by 10%, 20% and 30% sucrose in 0.1 M PBS sequentially. Coronal sections were prepared at 50 μm sliced with a freezing microtome (Yamato-Koki, Saitama, Japan). We divided the section into four. One in four consecutive sections was used for myelin stain, another for Nissl stain, and the other for observing DiI. All sections were mounted on gelatin-coated glass slides and air dried. The myelin staining was performed in the protocol described elsewhere (Pistorio et al., 2006). The Nissl staining was performed with thionin. The myelin and Nissl sections were dehydrated in graded ethanol solutions, immersed in xylene, and cover-slipped in DPX (Sigma-Aldrich Co., Buchs, Switzerland). Sections for observing DiI were cover-slipped in Immu-Mount (Thermo Fisher Scientific, Waltham, MA, USA). We followed previous nomenclatures for areal demarcation based on Nissl and myelin staining (Burman et al., 2011; Paxinos et al., 2012).

### Visual Stimuli

LightWave software (NewTek, San Antonio, TX, USA) was used to generate a stimulus set with various material appearances. We adjusted the surface reflectance properties on three dimensional object models on this platform and obtained a set of material stimuli that has surface property of seven material categories (ceramic, glass, hair, leather, metal, stone and wood), which were rendered on three distinct object models (bone, torus and amorphous). An additional category of objects was rendered with matte surface appearances, resulting in eight surface properties (termed “material category” for simplicity) and three shapes. Images labeled for a particular shape and material category in the stimulus set consisted of exemplars (3 or 4 images) that vary in color and/or circumference lighting condition. In addition, local pattern of surface roughness (in glass category), hair (fur), graining (wood), texture (leather and stone) were varied. Our aim was to put variations within each category so that we can capture a generalized selectivity for a particular category, but not the selectivity to a particular image. We also prepared pixel-shuffled images (shuffled stimuli) of each of the original material stimulus. The shuffled stimuli conserved outline (silhouette) shape and distribution of luminance and color of the original material stimuli, but local features important for our material perception (e.g., highlight, wood-graining, etc.) were largely destroyed. Neurons exhibiting identical selectivity for a particular material and the shuffled version of the same material were considered to be sensitive to the low-level pictorial properties mentioned above, but not to the material category. To assess the visual responses of the neurons, we presented visual stimuli on a 10-cd/m^2^ neutral gray background. The stimuli were ~8.5° in size, and were presented on a CRT display (SONY CPD-21DS2) located 57 cm away from the animal’s eyes. A contact lens was used to focus the eye contralateral to the recorded hemisphere at the display. The stimuli were presented monocularly at projected position of the fovea, identified using a retinoscope and a fundus camera. The presentation was in a pseudorandom order, with 400-ms stimulus durations interleaved by 600-ms inter-stimulus intervals.

### Data Analysis

Visual response of a multiunit was defined as the mean firing rate during a 400-ms visual stimulation period, with an 80-ms offset considering the signal delay in the brain, subtracted by the mean firing rate in a 400-ms pre-stimulus period immediately before the stimulus presentation. A multiunit was considered to be visually responsive if: (1) it reached minimum 3 spikes/s change in the firing rate between the pre-stimulus period and the stimulus evoked period; and (2) its response was statistically significant (*p* < 0.05, paired *t-test,* corrected for multiple comparisons using the Bonferroni method by the number of stimuli) of the responses was determined by comparing the firing rate in the visual stimulation period to that in the pre-stimulus period. Two-way ANOVA was performed to capture the dependence of each multiunit to surface material appearance and shape of the visual stimuli.

#### Computing Category Response

For multiunits with a significant visual response and a significant main effect of surface material category, its mean response to each material category (category response) was computed by averaging the response to 9 or 12 images (3 shapes and three or four exemplars varying in surface appearance) that belong to the same material category. Material preference of each multiunit was determined by comparing the magnitude of category responses across eight material categories. When average of the category response was computed across population, or across depths at each shank of the recording electrodes (depth-averaged category response), category response of each multiunit was normalized prior to the averaging by the SD of the firing rate in the 400-ms pre-stimulus period across all visual stimulus presentation trials.

Depth-averaged category responses were used for generating cortical category response magnitude maps (category response maps). Multiunits with insignificant visual response (*p* > 0.05 corrected, paired *t*-test) were included in the depth-average to reflect the density of responsive multiunit as well the selectivity of each multiunit to the response magnitude at each shank position. Similarity of the cortical response maps between material categories was quantified by computing the correlation coefficient between the category response maps.

#### Analysis of Gloss Preference and Shuffle Preference

To compare the preference of multiunits to the glossy (glass and metal) and the non-glossy (the rest of the stimulus material category) and the non-gloss stimulus images, we computed gloss selectivity index which was defined as (|*R*_G_| − |*R*_NG_|)/(|*R*_G_| + |*R*_NG_|), where *R*_G_ and *R*_NG_ are the mean responses to the gloss and the non-gloss images respectively. Shuffled-gloss selectivity index was defined as (|*R*_G−SH_| − |*R*_NG−SH_|)/(|*R*_G−SH_| + |*R*_NG−SH_|), where *R*_G−SH_ and *R*_NG−SH_ are the mean response to the shuffled versions of the gloss and the non-gloss images.

To compare the preference of multiunits to the shuffled and the original version of the preferred stimulus images, we computed shuffle selectivity index, which was defined as (|*R*_P−SH_| − |*R*_P_|)/(|*R*_P−SH_| + |*R*_P_|), where *R*_P_ and *R*_P−SH_ are the category response to the preferred material and the shuffled counterpart of the preferred material respectively. Histogram depicts the distribution of the shuffle indices excluding the data from monkey 2, to which shuffled stimuli were not presented.

#### Analysis of Functional Clustering

Spatial clustering of multiunits with similar category preference was quantified by computing the similarity of depth-averaged category response between all pairs of electrode shanks. The depth-averaged category response was a 16-element response vector (response to eight material categories and eight shuffled material categories) computed at the penetration site of each electrode shanks. The response similarity was a correlation coefficient of these response vectors computed between all pairs of electrode shanks where both shanks had one or more visually responsive multiunit. Coefficient values were considered significant when they exceeded the *p* < 0.05 statistical threshold for 16 data points.

Material preference maps were generated with multiunits that meet the following criteria: (1) visual response had a significant main effect of stimulus material category in the two-way ANOVA (material category and shape as factors); and (2) magnitude (absolute value) of the shuffle selectivity index is larger than a threshold value of 0.2 (corresponding to the preferred category having 1.5-fold larger mean visual response than its shuffled counterpart). Additional material preference maps were generated with different threshold values (0.1 and 0.0).

Spatial clustering of multiunits with preference for the glossy material was quantified by computing dispersion index defined as σ^2^/μ, where σ^2^ and μ are the variance and the mean of the gloss-selective multiunit count within a shank across 16 recording shanks. The dispersion index indicates spatial clustering of gloss-selective multiunits when >1, random distribution when ≈1, and spatial dispersion when <1. Statistical significance of the spatial clustering was determined against the hypothesis of random spatial distribution using the Pearson chi-squared goodness-of-fit test, where the dispersion index itself is the chi-square statistics with the number-of-shank −1 (= 16–1) degree of freedom.

#### Analysis of Image Statistics

Luminance and chromaticity of calibration images on the stimulus presentation monitor were measured using luminance/color meter (CS-100A, Konica Minolta, Tokyo, Japan). RGB pixel values of the stimulus images were converted to CIELAB color space (L*, a*, b*) using the calibration data. Pixel values were collected within the silhouette of each stimulus image to compute mean a* and mean b*, mean, variance, skewness and kurtosis of L* distribution. A MATLAB toolbox (Separable Steerable Pyramid Toolbox^1^) implementing the steerable pyramid by Portilla and Simoncelli (Portilla and Simoncelli, 2000) was used to filter images in two spatial scales and four orientations. Filtered outputs from different orientations were averaged to give non-oriented output for high and low spatial frequencies. Pearson’s correlation of these image statistics to neuronal response was computed for multiunits with selectivity for glass and metal material categories.

## Results

**Figure 1:**
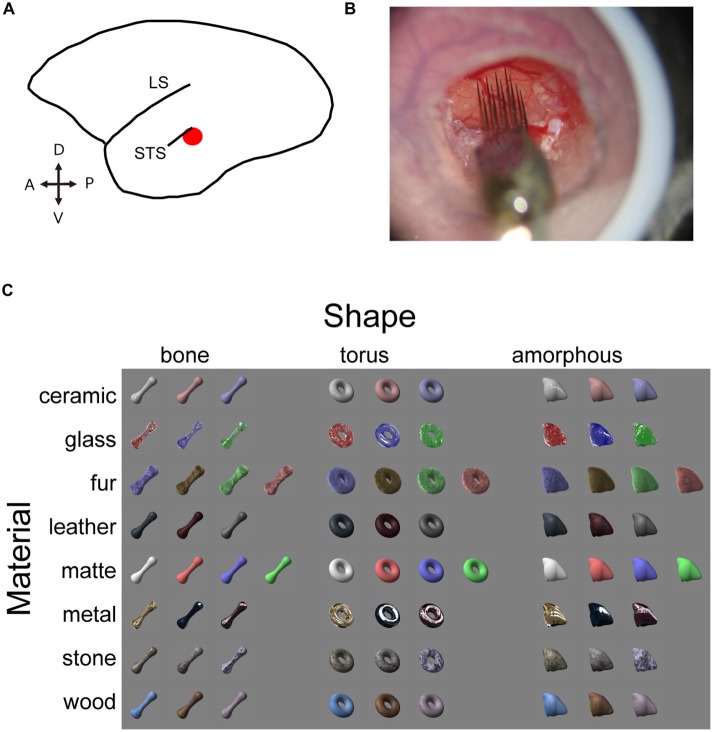
**Experimental design. (A)** Schematic illustration of marmoset brain. The target area is shown in red. **(B)** Electrophysiological recording with the silicone probe. Skull and dura are removed inside the chamber to expose the cortex. Agarose prevents the cortex from pulsating. An acryl plate with an opening for the silicone probe is fixed with a screwed cap but is removed in this picture for a clear view. **(C)** Visual stimulus set consists of eight material categories and three object shapes. Image pixels were further shuffled within the outlines to generate shuffled stimuli (see Figure 5 for an example).

In order to investigate the selectivity of neuron in the STS region (Figure 1A) for shapes and surface material category of objects, we presented a visual stimulus set (“material stimulus set”; Figure 1C) that vary both in surface material appearance (material: ceramic, glass, fur, leather, metal, stone, wood, or matte) and in three-dimensional shape (shape: bone, torus or amorphous) generated by a computer graphics software (LightWave) to marmoset monkeys maintained under anesthesia. We recorded multiunit neuronal signals from a cortical area posteroventral to the STS in four animals using multicontact linear-array electrodes. The arrays consisted of 4 or 16 shanks with a 400 μm horizontal spacing and eight recording sites in each shank with a 200 μm vertical spacing (Figure 1B). We recorded from a total of 143 visually responsive multiunits (*n* = 25, 18, 38 and 62 from 4 animals; *p* < 0.05, paired *t*-test, correction for multiple comparison with Bonferroni’s method; minimum 3 spikes/s change in the firing rate between the pre-stimulus period and the stimulus evoked period, see “Materials and Methods” Section for detail).

### Neurons with Selectivity for Glass and Metal Material Category in FSTv

**Figure 2:**
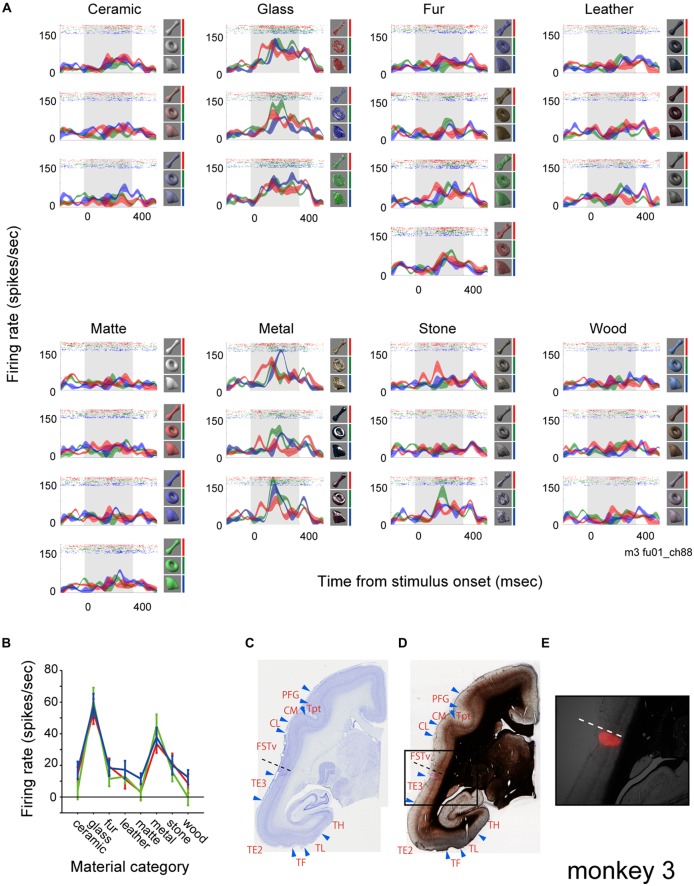
**Visual response of a representative multiunit. (A)** The visual response is shown as rastergrams and spike density functions (SDFs). Each panel depicts response from a surface material category. Rows of each panel depict response from a subtype of the surface material category. SDFs are shown as mean (colored lines) and standard errors (colored shadings). The colors of the rastergrams and SDFs indicate stimulus shape (red, bone; green, torus; blue, amorphous), and the corresponding stimuli are shown to the right of each panel. Gray shadings indicate the stimulus presentation period. **(B)** Tuning to the material category of the stimuli plotted for each shape (red, bone; green, torus; blue, amorphous). Error bars indicate standard errors. **(C–E)** Histological confirmation of the recorded area. Areal demarcations were drawn based on Nissl **(C)** and myelin** (D)** staining. Rectangle area in** (D)** is enlarged and superimposed with a fluorescent picture showing 1′-dioctadecyl-3,3,3′,3′-tetramethylindocarbocyanine perchlorate (DiI) labeling in **(E).** Dotted lines indicate the penetration site of the electrode shank from which the representative multiunit was recorded from (see Figure 4 for the positions of electrode penetration and DiI labeling).

Figure 2A shows rastergrams and spike density functions (SDFs) of the visually evoked activity of a representative multiunit. To analyze the effect of surface material category and physical shape of the visual stimuli, we applied a two-way ANOVA (with material category and shape as factors) to the visual response of the multiunit. The analysis exhibited a significant effect of the material category, but not the shape (*p* < 0.00001, 0.22 and 0.87 for the main effect of “material”, “shape” and their interaction respectively). This multiunit strongly responded to all nine visual stimuli that belong to the “glass” category and all that belong to the “metal” category regardless of the stimulus shape (Figure 2A). It responded mildly to some stimuli that belong to the “fur” and “stone” categories, but scarcely to the other surface material categories (Figure 2A). Penetrating position of the recording electrode was confirmed by marking a subgroup of shanks of the 16-shank (128-channel) array with DiI, and referring the DiI fluorescence (Figure 2E) with the histologically defined borders (Figures 2C,D). The shank from which the representative multiunit was recorded (Figures 2C,D, dotted lines) was found to lie in an anatomically defined area FSTv (suggested homolog of areas PGa and IPA of macaque monkeys; Solomon and Rosa, 2014).

**Figure 3:**
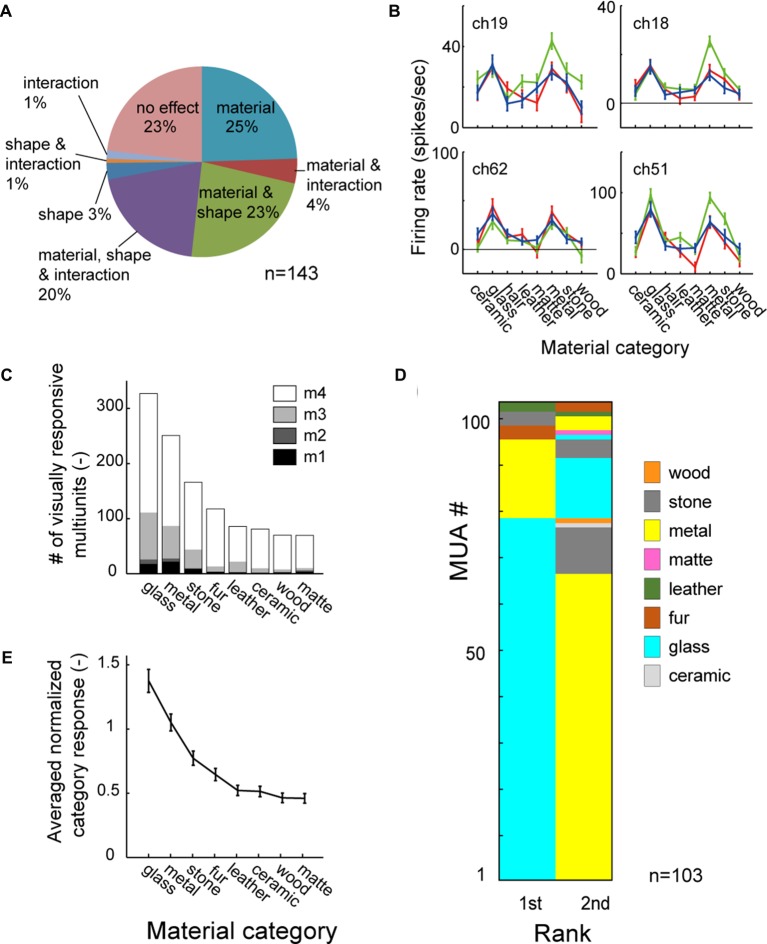
**Material category preference pooled across population. (A)** Result of two-way analysis of variance (ANOVA) for material category and shape shown in percentage. **(B)** Representative tuning curve of multiunits with significant main effect (*p* < 0.05) of both material and shape. Multiunits in right column also have significant interaction (*p* < 0.05). Color conventions are same as Figure 2B. **(C)** Mean category response averaged across all material-sensitive multiunits. **(D)** 1st and 2nd best (preferred) category are shown in color-code for all material-sensitive multiunits (*p* < 0.05, main effect of material). **(E)** Number of stimulus images in each material category that evoke significant visual response to the multiunits. Count is pooled across the material-sensitive multiunits. The counts of fur and matte category are corrected for the difference in the number of stimuli by multiplying with 3/4 (see “Materials and Methods” Section).

Figure 3A shows the population result of the two-way ANOVA. Within the 143 visually responsive multiunits, 29% (10/25, 4/18, 17/38 and 10/62 from 4 animals) had significant main effect (*p* < 0.05, two-way ANOVA) only of the material, 3% (0/25, 3/18, 0/38 and 2/62) only of the shape. 43% (5/25, 3/18, 16/38 and 38/62) had significant main effect of both the material and the shape (representative tuning curves shown in Figure 3B). Thus, up to 72% (15/25, 7/18, 33/38 and 48/62) of the population had main effect for the surface material category, and were termed as “material-sensitive multiunits” in the present study. A significant interaction (*p* < 0.05, two-way ANOVA) between the two factors was found in 26% (2/25, 0/18, 8/38 and 28/62) of the visually responsive multiunits (Figure 3A). Friedman’s test, a nonparametric two-factor analysis exhibit a similar result; 31% (10/25, 2/18, 22/38 and 10/62 from 4 animals) had significant main effect (*p* < 0.05, Friedman’s test) only of the material, 3% (0/25, 3/18, 0/38 and 2/62) only of the shape, and 41% (5/25, 5/18, 11/38 and 38/62) of both the material and the shape.

To investigate whether the simultaneous selectivity for the glass and the metal categories found in the representative multiunit (Figure 2) is preserved across the population, we analyzed a rank order of material category preference in all of the material-sensitive multiunits (Figure 3D). Of the 103 material-sensitive multiunits, 76% (78/103) had glass and 17% (17/103) had metal as the most preferred category. Of those with primary preference for the glass category, 85% (66/78) had metal as the second preferred category. Of those with primary preference for the metal, 76% (13/17) had glass as the second preferred category. Altogether, glass and metal occupied the 1st and 2nd ranks of the category-preference rank order in 77% (79/103) of the material sensitive multiunits.

Furthermore, in a count of stimulus images that elicit significant visual response pooled across the population (Figure 3C), the glass and metal were the 1st and 2nd most preferred category. The glass and metal categories also elicited 1st and 2nd largest population average of category response pooled across animal (Figure 3E). Thus the preference for both the glass and metal categories is a common property observed among large portion of neurons in the FSTv.

### Selectivity to Material Category by Population Neural Activity

**Figure 4:**
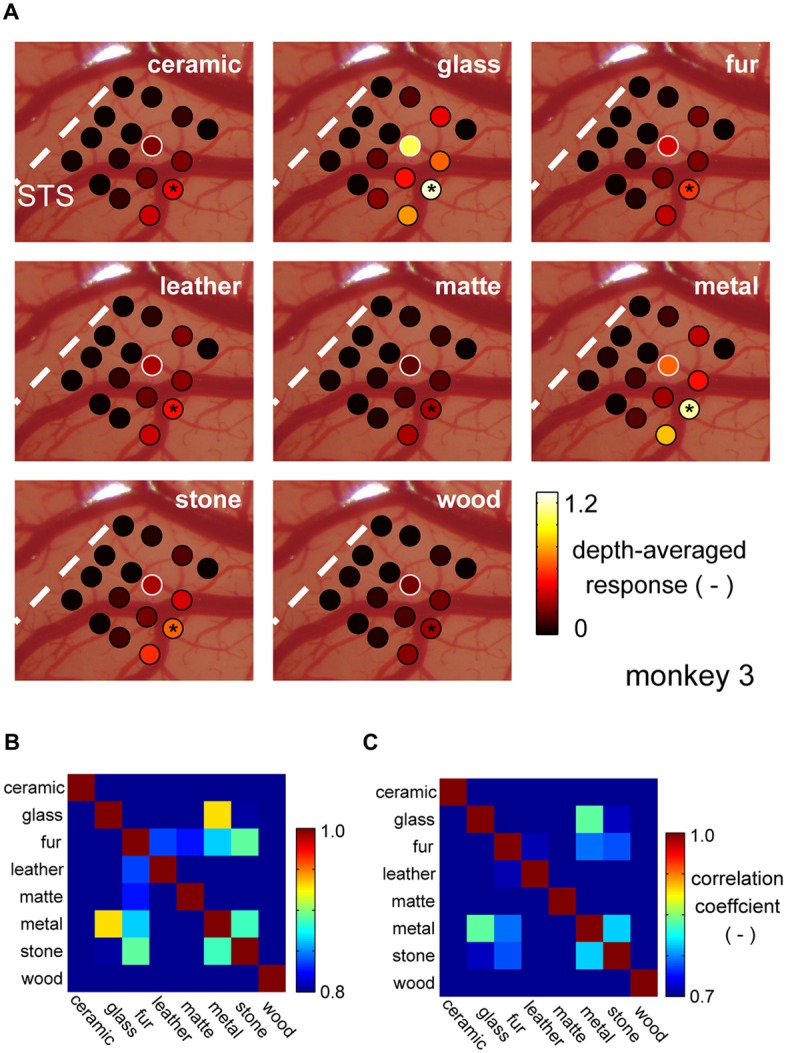
**Cortical activation pattern representing material categories. (A)** Cortical activation pattern across material categories. Patches represent locations where individual shanks of the recording electrode penetrated. Each shank has multiple recording sites across depths. Color of the patches represents the magnitude of the multiunit response averaged within stimulus category and across depths. The visual response of each multiunit was normalized by its spontaneous activity before the averaging (see “Materials and Methods” Section). White patches indicate the position where the representative multiunit was recorded from. Black asterisk indicates the position of DiI labeling. **(B,C)** Similarity of the response pattern across material categories computed with depth-averaged response pattern **(B)** and individual multiunit response pattern** (C)**.

It is commonly said that there is population coding (coding by pattern of activity) in the higher visual cortices. To address whether the common selectivity for the glass and the metal over the other categories is robustly represented by population, we generated cortical activation maps for each material category, generated by averaging normalized category responses at each electrode shanks. Figure 4A depicts category response maps of a representative animal (monkey 3). Regions that strongly responded to glass images seemed to show strong response to metal images as well, but less so to the images of other surface material category.

To evaluate this similarity of the category response maps, we computed a correlation coefficient (*r*) between the maps all category pairs. Figure 4B is a matrix of coefficient values averaged across animal. Highest correlation was found between the response maps of the glass and metal category (Figure 4B). Correlation computed between the original (not depth-averaged) multiunit category response patterns was also the highest between the glass and metal categories (Figure 4C). These results suggest that there is a group of neuron in the area FSTv of marmoset monkeys that are selective to a common visual entity found in “glass” and “metal” images, which may be glossiness of the image. Thus, we combine the glass and metal category images from hereon, and refer to as “gloss” images.

### Effect of Pixel Shuffling of the Stimulus Image

**Figure 5:**
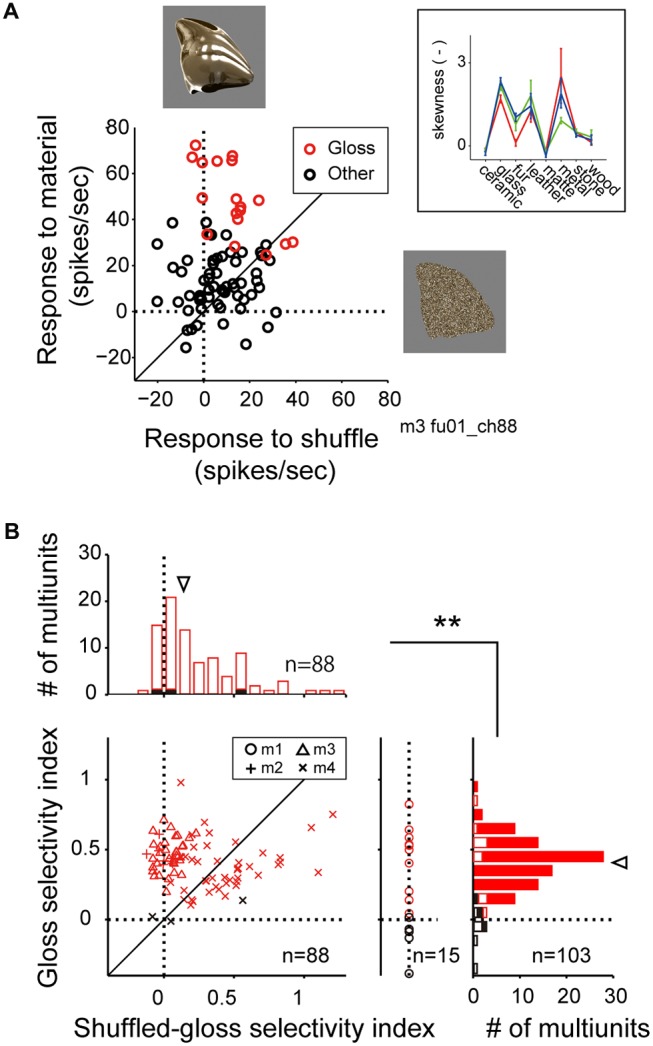
**Comparison of visual response to the material stimuli and the shuffled stimuli. (A)** Scattergram showing visual response magnitudes evoked by the normal (ordinate) and the shuffled (abscissa) version of the stimulus images. Red makers depict the response to the stimulus images that belong to the glossy (“glass” “metal”) categories. Black markers depict the response to the images that belong to the other categories (“ceramic”, “fur”, “leather”, “matte”, “stone” and “wood”). Stimulus images are exemplars of the metal category **(top)** and the shuffled version of the same exemplar **(right)**. **(Inset)** Category-wise skewness of image luminance histogram. Color conventions are same as Figure 2B. **(B)** Histograms depict the distributions of the gloss selectivity indices (**right**; see “Materials and Methods” Section), and the shuffled-gloss selectivity indices **(top)**. Color of the markers and bars indicate the significant (red) or insignificant (black) sensitivity to material category. Closed bars in the histograms indicate data from monkeys 2, 3 and 4. Open bars in **right** indicate data from monkey 1 that lack data elicited by the shuffled stimuli. Inverted arrowheads indicate median. **p < 10^−7^, Mann-Whitney *U* test. Median and statistical test computed for the population pooled across multiunits with significant (red) or insignificant (black) sensitivity to material category.

Perception of surface gloss is known to relate to (Motoyoshi et al., 2007), but cannot be fully explained by (Anderson and Kim, 2009; Kim and Anderson, 2010), skewness of luminance histogram of incoming images. The glossy image categories (glass and metal) in our stimulus set indeed had highly skewed luminance histogram (Figure 5A, inset). To evaluate whether the selectivity of the multiunit to glossy material categories are actually the selectivity to the positive skew (i.e., bias to dark pixel with long-tailed distribution to bright pixel), we generated a pixel-shuffled version of our material stimulus set (“shuffle stimulus set”). Pixel shuffling dramatically changed perceptual appearance of the stimulus set, but maintained many of the low-level visual features. In the representative multiunit, preference for the glossy images found in the response pattern to the original material stimulus set was lost in the response pattern elicited by the shuffled counterpart (Figure 5A, red markers). Thus, mere skew of the image luminance pattern could not fully explain the selectivity of the multiunits that we termed gloss-selective.

For the material-sensitive multiunits (*p* < 0.05, main effect of material category in two-way ANOVA; *n* = 103), we computed gloss selectivity index (see “Materials and Methods” Section for definition) to quantify their selectivity for the glossy material categories (glass and metal). A large population of the material-sensitive multiunits had positive gloss selectivity index (median = 0.41; Figure 5B, right histogram, arrowhead; positive in 96 out of 103 multiunits), suggesting that the preference for glossy image is a widely common property found in the FSTv neurons. For the material-sensitive multiunits recoded from monkeys 2, 3 and 4 (*n* = 7, 33 and 48), we computed shuffled gloss selectivity index, which is an equivalent of the gloss selectivity index but quantifies the selectivity for the shuffled version of the gloss category over the other shuffled categories (Figure 5B). The shuffled gloss selectivity indices were positive for a large portion of the population (*p* < 10^−9^, binomial test; median = 0.14; Figure 5B, top histogram, arrowhead; positive in 72 out of 88 multiunits), but were overall smaller than the gloss selectivity indices computed from the original material category set (*p* = 0.000002, Mann-Whitney *U* test).

In the scattergram of gloss selectivity index (ordinate) vs. the shuffled gloss selectivity index (abscissa; Figure 5B), a large group of neuron from monkey 4 (30 out of 48) was distributed below the diagonal, suggesting variability in the population recorded from this animal. However, a substantial number of multiunits from all three animals tested (7, 33 and 18) were distributed above the diagonal. These results support the notion that there are FSTv neurons selective to the image glossiness, and not on other image parameters such as silhouette shape, or mean and distribution of luminance and color unaffected by pixel shuffling procedure.

### Gloss-Selective Neuron Cluster in FSTv

**Figure 6:**
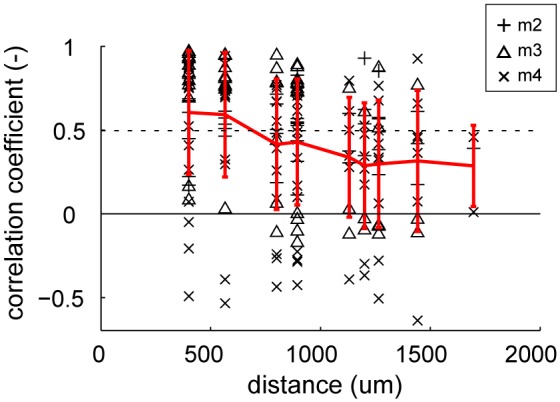
**Similarity of stimulus selectivity between depth-averaged visual responses recorded in different electrode shanks.** Correlation coefficients were computed between pairs of depth-averaged response to 16 categories (8 material category and 8 shuffled material category) from 16 shanks (see “Materials and Methods” Section for detail). The coefficient values are plotted against the horizontal distance between the shanks. The individual coefficient values (black markers) and their mean (red lines) are indicated in the plot. Error bars indicate standard deviation (SD). Dotted horizontal line indicates the statistically significant threshold (*r* = 0.50, *p* < 0.05 for 16 categories).

An important question was whether these gloss-selective neurons are organized in a cluster in marmoset FSTv, as the gloss-selective neuron in other species (Hiramatsu et al., 2011; Nishio et al., 2012; Okazawa et al., 2012). First, we investigated whether multiunits with similar category preference cluster along the cortical plane by comparing the similarity of depth-averaged category responses to the distance of the recorded electrode shanks (Figure 6). The mean correlation coefficient value of the depth-averaged category response (response to eight material categories and eight shuffled material categories) exceeded chance level (*r* = 0.50, *p* < 0.05) when the distances between the recorded electrode shanks were 566 μm or closer. This result supports that neurons in area FSTv functionally cluster by their preference for material category.

**Figure 7:**
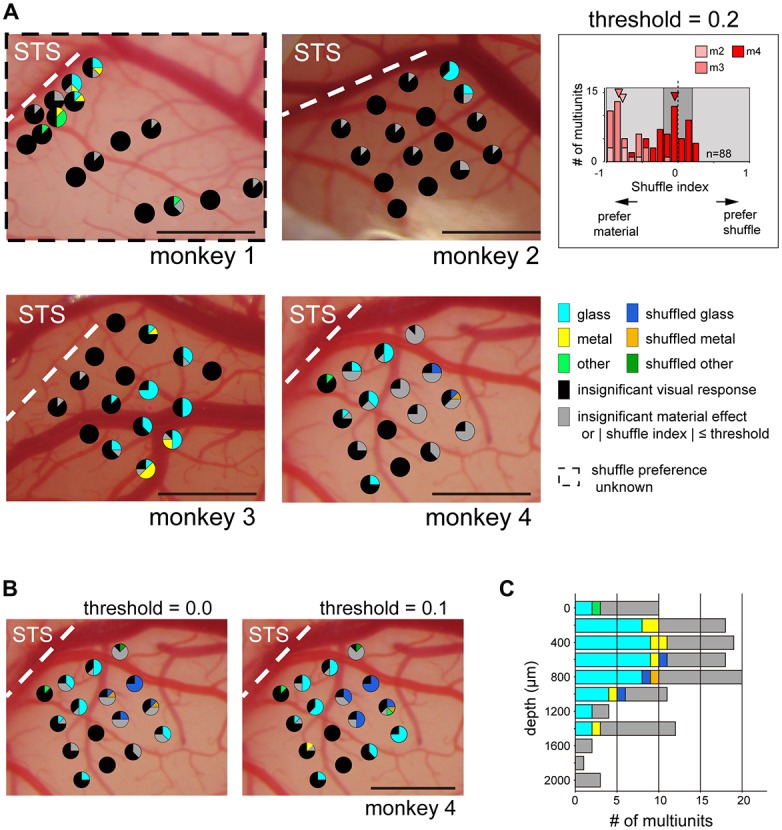
**Category preference map in ventral part of the fundus of the superior temporal area (FSTv).** Pie chart represents proportion of multiunits within an electrode shank that preferred the category specified by the color code. Multiunits with sensitivity for the material category (*p* < 0.05, two-way ANOVA) and with absolute shuffle index value above threshold (|shuffle index| > threshold, light gray background in **inset**) were used to generate the map. The latter criterion was not applied to monkey 1 because the shuffled stimuli were not presented to this animal. Pie charts locations follow the convention of Figure 6. **(A)** Category preference map of all animals. Threshold value was set to 0.2, corresponding to the preferred category having 1.5-fold larger visual response than its shuffled counterpart. **(Inset)** Distribution of the shuffle indices of all animals excluding monkey 1. Arrowheads indicate the median of the index values of each animal. **(B)** Category preference map of monkey 4 with different threshold values. Note that multiunits with preference to glossy stimuli (cyan: glass, yellow: metal) and those with preference to the shuffled version of the glossy stimuli (blue: shuffled glass, orange: shuffled metal) are localized in segregated manner regardless of the threshold value. **(C)** Depth profile of category preference pooled across all shanks in monkeys 2–4. Criteria and the threshold are identical to those in **(A)**. Penetration depth differed across animal (2000 μm, monkey 2; 1600 μm, monkey 3; 1400 μm, monkey 4). Scale bars: 1 mm.

Next, we directly investigated the clustering of multiunits selective to glossiness of the image. For monkeys 2–4 presented with the shuffled stimulus set, we mapped the cortical surface by the material and shuffle preference (Figure 7A, glass, shuffled-glass, metal, shuffled-metal, other, or shuffled-other material). We selected multiunits that: (1) have significant sensitivity to the surface material category (*p* < 0.05, main effect in two-way ANOVA); and (2) have stronger preference for either the original or the shuffled version of the material category images. Shuffle selectivity index (see “Materials and Methods” Section) evaluated the preference for the shuffled stimuli; negative (positive) value indicates preference for the original (shuffled) version of the preferred material category. Majority of the tested units had negative shuffle index (*p* < 10^−7^, binomial test; 7/7, 33/33 and 30/48 respectively in monkeys 2–4, Figure 7A inset). In fact, all the visually responsive multiunits from monkey 2 and 3 had negative shuffle selectivity index (responded larger to the preferred material category than to its shuffled counterpart, Figure 7A inset), regardless of its preferred material category. On the other hand, 38% (18/48) of the material-sensitive multiunits from monkey 4 had positive shuffle selectivity index (i.e., responded smaller to the preferred stimulus category than to its shuffled version, Figure 7A inset), corresponding to the variability in the preference for shuffled stimuli across animal seen in Figure 5B.

The maps from monkeys 2–4 showed that majority of the multiunits with preference to the glass (Figure 7A, cyan) or the metal (Figure 7A, yellow) categories over the other categories (Figure 7A, light green) are confined in a subset of electrode shanks in a spatially packed manner (monkey 2: seven multiunits in two adjacent shanks, monkey 3: 33 multiunits in nine adjacent shanks, monkey 4: 10 multiunits in four adjacent shanks, but two more multiunits in a separate shank; Figure 7A). To quantify the clustering of the gloss-selective multiunits, we computed dispersion index by the count of gloss-selective multiunit per shank. It indicates spatial clustering when >1, random and independent distribution when ≈1 and spatial dispersion when <1 (see “Materials and Methods” Section). Dispersion indices were 4.0 (*p* < 10^−6^, Pearson chi-squared goodness-of-fit test comparing with random distribution; monkey 2), 2.5 (*p* < 0.001; monkey 3) and 2.2 (*p* < 0.01; monkey 4) respectively, showing significant spatial clustering in all cases.

We also mapped the cortical surface of monkey 1 by the material preference of the multiunits without considering their preference for the shuffled stimuli (Figure 7A). There were more multiunits with preference for the two glossy categories (six and four multiunits for glass and metal respectively) than the six other categories combined (five multiunits), and multiunits with putative gloss-selectivity were spatially clustered (10 multiunits in four adjacent probes; dispersion index = 2.5, *p* < 0.001). Interestingly, the subset of multiunits with a preference for the shuffled stimuli (shuffle selectivity index > 0.2; Figure 7A inset, light-gray area in the right half of the histogram) responded best to the shuffled glass (Figure 7A, blue in monkey 4; *n* = 3) or to the shuffled metal (Figure 7A, orange in monkey 4; *n* = 1) categories. Moreover, these multiunits formed an exclusive cluster of its own (four multiunits in two adjacent shanks; dispersion index = 1.9, *p* < 0.05; none of seven other visually responsive multiunits recorded from these shanks preferred the glossy stimuli). This tendency did not change even if we lowered the threshold value to 0.1 (Figure 7B, left; 12 multiunits in four adjacent shanks preferred the shuffled gloss; dispersion index = 3.5, *p* < 10^−5^; no other visually responsive multiunits in these shanks preferred the glossy stimuli), or to 0.0 (Figure 7B, right; 16 multiunits in four adjacent shanks preferred the shuffled gloss; dispersion index = 3.7, *p* < 10^−5^; no other multiunits preferred the glossy stimuli).

Figure 7C shows depth-profile of the material preference of multiunits recorded in monkeys 2, 3 and 4. Gloss-selective existed across depths (0–1400 μm cortical depth) uniformly (approximately 40%–60% of the visually responsive multiunits in 200–1200 μm depth preferred glass or metal).

## Discussion

The primate ventral visual cortex prepares specialized functional architecture for representing critically important information, such as face (Desimone et al., 1984; Kanwisher et al., 1997; Tsao et al., 2003; Hung et al., 2015a), body (Pinsk et al., 2005), place (Epstein and Kanwisher, 1998) and color (Tootell et al., 2004; Conway and Tsao, 2006). Recent studies have found neural structures representing gloss in human (Hiramatsu et al., 2011) and macaque (Okazawa et al., 2012; Goda et al., 2014) ventral visual cortices. In this study, we demonstrated for the first time that neural cluster specialized for glossy surface exists as well in common marmoset FSTv, a ventral subregion of FST. This result suggests that surface glossiness is a perceptual feature critically important to be maintained across very large difference in species, and presumably across evolutionary time required for the separation of the species. This is not surprising considering the importance of gloss-perception, which contribute to identifying water surface (Meert et al., 2014), differentiating object from their surface property (Landy, 2007), and estimating 3D shape of objects (Fleming et al., 2004). With its lissencephalic form, marmoset visual cortex may be an ideal target for future studies combining anatomical identification and physiological characterization of the cortical network representing gloss information *in vivo*. It would provide us an excellent platform for tackling the mechanisms that transform the material-related visual information from image-based to perceptually categorized form.

### Gloss-Selective Neuron in FSTv

In macaque monkeys, FST has been described as a region that receive strong direct input from a motion-selective area, MT (Boussaoud et al., 1990; Felleman and Van Essen, 1991; Kravitz et al., 2013), and is reported by Mysore et al. (2010) that some neuron here are selective to “3D structure-from-motion” (3D depth perceived from particular motion of surface patch textured with random dots. However, Kaas and Morel (1993) reported in a study with New World monkey (owl monkey) that in terms of connection, FST can be subdivided to dorsal FST (FSTd) receiving strong inputs from the dorsal visual areas including area MT and to FSTv receiving strong inputs from the ventral visual areas but not from the dorsal visual areas. They concluded that FSTv is a part of the ventral visual stream, whereas FSTd is a part of the dorsal visual stream, consistent with the description of macaque monkey FST (Boussaoud et al., 1990). Given the limited area of mapping in the present study, we need further investigation to determine what other sites are involved in the representation of glossy surface. However, our finding of gloss-selective area in marmoset FSTv, a region considered as part of the ventral visual network, matches the previous result of the whole-brain analysis in macaque that reported gloss-processing network lies within the ventral visual area (Okazawa et al., 2012). It seems worthwhile to note that being at the border to the dorsal visual stream, FSTv is also located in a good position to receive information of in-depth rotation, which is known to help perceive the surface “shininess” (glossiness) of objects (Doerschner et al., 2011).

### Clustering of Neurons in FSTv and their Possible Relevance in the Representation of Gloss

Neurons in FSTv were found to cluster by their selectivity to the material category. Significant level of inter-shank correlation for material selectivity was found when the inter-shank distance was ≈500 μm or smaller. This roughly matches the size of the functional column selective to other’s reaching action recently found in FSTv (400–800 μm, Suzuki et al., 2015b), and those in other higher visual cortices (400–600 μm, Albright et al., 1984; Fujita et al., 1992).

In all three animals tested with the shuffled image set, we found cluster of neurons with preference for the image glossiness, but not on other low-level image parameters including skewness of luminance distribution. Gloss-selective clusters were located immediately below STS, except in monkey 3 where the cluster was shifted more ventrally. This animal had an exceptionally shallow STS (Figures 2C–E), which may have resulted in ventral shifting of the STS ventral lip. Such inter-animal variation of the STS depth has been described previously, ranging from 2.5 mm to “not more than a mild depression” (de la Mothe et al., 2006). Furthermore, we found an additional cluster in monkey 4 that shows selectivity to the shuffled version of the gloss images. Here the shuffled-gloss-selective cluster was located adjacent and caudal to the gloss-selective cluster, forming a population with an exclusive selectivity (i.e., no multiunit within the penetration site had preference for the normal glossy material). It is known that neuron with opposing preference can sharpen the tuning of each other, when connected with inhibitory network (Bonds, 1989). Thus, the potential function of the shuffled-gloss-selective cluster could be to form selectivity for gloss information beyond low-level visual statistics through inhibitory connection from the shuffled-gloss-selective cluster to the gloss-selective cluster. This speculation still needs further investigation with causality analysis and activation/inactivation experiments, and more importantly additional observations from more animals.

### Statistical Property of the Stimulus Images Contributing to Neuronal Selectivity

**Figure 8:**
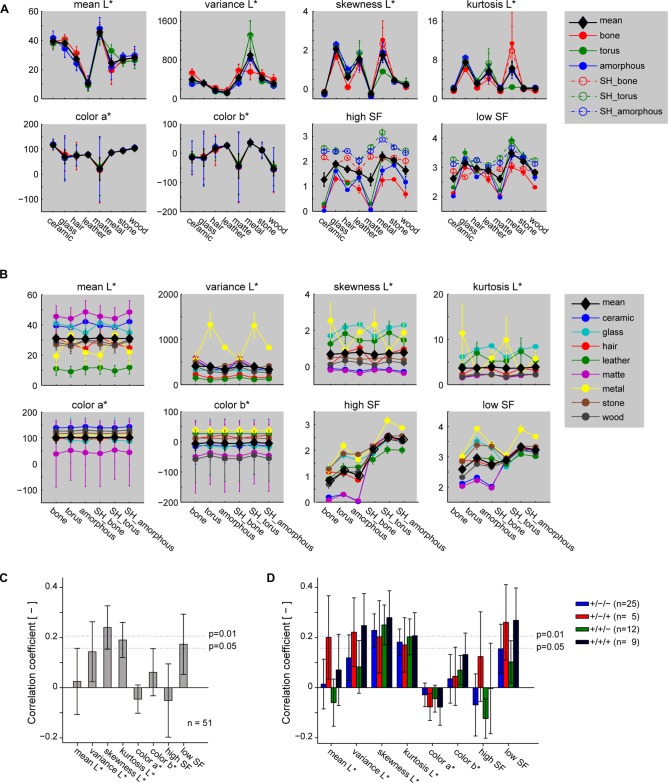
**Statistics of the stimulus images and their relation to neuronal selectivity. (A,B)** Image statistics of the stimulus images sorted by material category **(A)** or shape **(B)**. L*, a*, and b* represent lightness and color in CIE Lab color space. Statistics of the shuffled stimuli (labeled “SH”) are shown as a separate plot in **(A)**, but not in** (B)** for display reasons. **(C)** Correlation of the image statistics to response of the multiunits that prefer the glossy categories (shown in cyan and yellow in Figure 4A, monkeys 2–4). **(D)** Correlation of the image statistics to the response of the same multiunits in **(C)**, but shown separately by their significance of shape main effect and interaction. “+” and “−” signs in the legend depict significance for “material main effect”/“shape main effect”/“interaction” (two-way ANOVA) in the indicated order. Error bars indicate standard errors for **(A,B)**, SDs for **(C,D)**.

In this study, we investigated the preference of neurons to gloss visual images based on their preference to glass and metal images we generated by adopting the surface property for these categories inferred in the LightWave software. The resulting images of metal and glass had highly specular surface that elicit sense of glossiness. To investigate whether response of the gloss-selective neurons could be explained by basic image statistics, we computed mean, variance, skewness and kurtosis of the luminance distribution, two levels of spatial frequency (SF), and colors in the CIELAB color space (Figure 8). Skewness and kurtosis of the luminance, and low SF had local peaks for glass and metal categories (Figure 8A). However, none of the parameters seemed sufficient to explain the properties of the gloss-selective neurons because they are either not attenuated at all, or only partly attenuated by pixel shuffling. These parameters indeed showed significant, but only weak association to the response of gloss-selective multiunits (Figure 8C; *p* < 0.01, *R* = 0.24 for skewness; *p* < 0.05, *R* = 0.19 for kurtosis; *p* < 0.05, *R* = 0.17 for low SF. See Figure 7A, monkeys 2–4 for selection of gloss-selective multiunits). Other physical parameters that characterize surface reflectance, namely diffuse reflectance, specular reflectance and spread of the specular reflectance, cannot be retrieved from LightWave. Thus, quantitative correspondence of the gloss-selective neural activity in marmoset FSTv to these physical parameters related to gloss is unknown. Similarly, psychological parameters related to distinctness of gloss and contrast gloss, both of which can be computed by from the physical parameters of reflectance (Ferwerda et al., 2001), is subject to a further study.

### Shape Sensitivity of the Gloss-Selective Neurons

The visual stimulus set used in this study varies in the three-dimensional shape as well as in the surface material category. Within the visually responsive population of multiunits we recorded from, those with a main effect only for shape was scarce (4%), whereas those with a main effect only for material (29%) was found in a much larger portion. However, some material sensitive multiunits had additional sensitivity to shape in either linear (23%, main effect of both material and shape) and/or non-linear (20%, main effect of material and interaction between material and shape) manner. For example, representative multiunits with shape main effect (Figure 3B) and interaction (Figure 3B, right column) had tuning curves with elevated peaks for the torus-shaped metal category.

One possibility is that this subpopulation of neuron responded to local highlight structure specific not only to the material, but also to the shape of the stimulus. In fact, image statistics such as low SF and variance that would yield high values from highlights in the image also yielded elevated values for the torus-shaped metal (Figure 8A, green lines; Figure 8B, yellow lines). Moreover, mean correlation of neuronal response to the variance and lows SF of stimuli exceeded significance threshold (*p* < 0.01, Figure 8D, red and black bars) for multiunits with significant interaction effect of the material and shape, but not for those without significant interaction (*p* > 0.05, Figure 8D, blue and green bars). It suggests at least partial contribution of these image statistics to the subpopulation of gloss-selective multiunits with significant interaction to the material and shape. On the other hand, variance and low SF clearly differ from the property of gloss-selective multiunits defined in the present study in that they do not resemble the >0.5-fold drop of gloss-selectivity by image shuffling (Figure 7A inset). Furthermore, images labeled for particular shape and material in our stimulus set consists of three or four exemplar images that vary in circumference lighting condition, giving variation to the shape and position of the local highlights. Thus, neither of these image statistics seems reasonable for fully explaining the property of gloss-selective neurons defined in the present study, especially those without significant interaction of material and shape of the stimuli (*n* = 37/51, Figure 8D, blue and green bars). Nevertheless, considering the close location of the gloss-selective neurons, it would be possible that the output of the neurons with sensitivity to local highlights are pooled to contribute to the selectivity of neurons with sensitivity to gloss images irrespective of image shape and highlight location.

## Author Contributions

NM, TB, WS and NI designed the study. TB generated the CG stimuli. NM, TB and HA performed the analysis. All authors participated in the experiments and the data interpretation. NM wrote the manuscript.

## Funding

Japan Society for the Promotion of Science (JSPS) KAKENHI JP25871171 and JP16H01683 to NM; JSPS KAKENHI JP22135007 and Brain Mapping by Integrated Neurotechnologies for Disease Studies (Brain/MINDS) from Japan Agency for Medical Research and development (AMED) to NI.

## Conflict of Interest Statement

The authors declare that the research was conducted in the absence of any commercial or financial relationships that could be construed as a potential conflict of interest.

## Footnotes

^1^https://www.mathworks.com/matlabcentral/fileexchange/43909-separable-steerable-pyramid-toolbox
